# Comprehensive Review of Anesthetic Evaluation and Management in Obese Female Patients Undergoing In Vitro Fertilization

**DOI:** 10.7759/cureus.47521

**Published:** 2023-10-23

**Authors:** Shubham Petkar, Vivek Chakole, Aishwarya Nayak

**Affiliations:** 1 Anaesthesiology, Jawaharlal Nehru Medical College, Datta Meghe Institute of Higher Education and Research, Wardha, IND

**Keywords:** reproductive medicine, bariatric anesthesia, infertility, anesthetic management, in vitro fertilization (ivf), obesity

## Abstract

Obesity has become a global health epidemic with profound implications for various medical specialties, including reproductive medicine. This comprehensive review focuses on the anesthetic evaluation and management of obese patients undergoing in vitro fertilization (IVF) procedures. Obesity, as defined by BMI, is associated with infertility and poses unique challenges for anesthetic care. The review also addresses the timing of anesthesia concerning IVF procedures, the impact of obesity on IVF success rates, and the importance of emotional and psychological support for obese patients undergoing IVF. Challenges and future directions in the field are highlighted, focusing on ongoing research, emerging technologies, and the role of multidisciplinary teams in managing these complex cases. In conclusion, this review underscores the critical role of tailored anesthesia and perioperative care in optimizing outcomes for obese patients undergoing IVF. It provides valuable insights for anesthetic providers, reproductive specialists, and healthcare teams, emphasizing the need for a patient-centered approach to address the unique challenges posed by obesity in the context of assisted reproductive technology.

## Introduction and background

Obesity has emerged as a global health epidemic, affecting millions of individuals worldwide and presenting a significant public health challenge. In 2005, approximately 1.6 billion adults globally were overweight (BMI: 25-30 kg/m2), and at least 400 million were obese (BMI: >30 kg/m2). By 2015, these numbers had escalated to 2.3 billion and 700 million, respectively [1]. The implications of obesity extend beyond general health, influencing specialized medical procedures. One such area where the impact of obesity is particularly notable is in reproductive medicine, especially concerning in vitro fertilization (IVF). IVF offers hope to couples struggling with infertility, yet its success can be profoundly affected by the unique challenges posed by obesity [1-3]. Obesity is defined by an excess of adipose tissue and specific BMI criteria. As obesity rates continue to rise, it has become increasingly common to encounter obese patients seeking IVF to fulfill their desire for parenthood. This intersection of obesity and IVF brings to the forefront a range of complex medical and logistical considerations, including the administration of anesthesia. Understanding and addressing these considerations is pivotal to ensuring the safety and efficacy of IVF procedures in this patient population [4]. The rationale behind delving into the anesthetic aspects of IVF in obese patients is multifaceted. First and foremost, obesity is a known risk factor for infertility, creating a significant patient population that necessitates specialized care. Secondly, IVF is an intricate and delicate procedure that requires precise management, from oocyte retrieval to embryo transfer. This necessitates understanding how obesity affects various aspects of anesthesia and perioperative care [5]. Moreover, obesity presents unique challenges in airway management, drug dosing, and perioperative complications, all of which are paramount to the safety and success of IVF. Inadequate attention to these considerations can lead to suboptimal outcomes, including anesthesia-related complications, reduced success rates in IVF, and increased morbidity for the patient [6]. This comprehensive review aims to provide an in-depth analysis of the anesthetic evaluation and management of obese patients undergoing IVF procedures. This review aims to shed light on the various facets of this multifaceted issue, encompassing the medical intricacies and the emotional and psychological aspects of care. Throughout this review, we will examine the complexities obesity introduces to IVF, ranging from initial preoperative assessments to intraoperative and postoperative considerations. We will delve into the nuances of anesthetic choices, equipment adaptations, medication dosing, and ventilation strategies tailored for obese patients undergoing IVF. Additionally, we will consider the timing of anesthesia in IVF procedures, its subsequent impact on success rates, and the vital role played by multidisciplinary teams in delivering comprehensive care.

## Review

Obesity and IVF: an overview

Definition of Obesity and Its Classification

Obesity is a complex health condition marked by excessive adipose tissue accumulation in the body. BMI is the most commonly used metric for defining and categorizing obesity, as classified by the WHO in Table 1.

**Table 1:** BMI classifications for obesity. Source: The table is taken from open source [7].

BMI Category	BMI Range
Underweight	BMI <18.5 kg/m^2^
Normal Weight	BMI 18.5-24.9 kg/m^2^
Overweight	BMI 25-29.9 kg/m^2^
Obesity Class I (Moderate Obesity)	BMI 30-34.9 kg/m^2^
Obesity Class II (Severe Obesity)	BMI 35-39.9 kg/m^2^
Obesity Class III (Morbid Obesity)	BMI ≥ 40 kg/m^2^

Prevalence of Obesity and Its Association With Infertility

The global prevalence of obesity has witnessed a concerning and continuous rise in recent decades, emerging as a significant public health issue. Research indicates that this surge in obesity rates has far-reaching consequences, including its profound impact on reproductive health, both in men and women. The association between obesity and infertility is a growing concern within the medical community, as it sheds light on the intricate interplay between body weight and reproductive function [8]. In women, obesity has been linked to a myriad of reproductive challenges. Notably, it is associated with irregular menstrual cycles, often stemming from hormonal imbalances. It can lead to conditions such as anovulation, wherein ovulation fails to occur, and polycystic ovary syndrome (PCOS), a common endocrine disorder characterized by multiple cysts on the ovaries. These factors collectively contribute to the difficulty obese women may encounter in conceiving naturally. Women with BMIs between 24 and 31 exhibit a 30% higher prevalence of anovulation-related infertility than their normal-weight counterparts. For those with a BMI exceeding 31, the likelihood of such infertility rises by an astonishing 170% [9]. Obesity's influence extends beyond the female reproductive system, affecting male fertility. Obese men experience reduced sperm quality, characterized by alterations in sperm morphology and lower sperm motility. Furthermore, hormonal imbalances associated with obesity can compromise the male reproductive function [10]. Notably, the impact of obesity on fertility is not confined to individual cases. In couples where one or both partners are obese, the likelihood of subfertility, defined as a prolonged time to achieve pregnancy despite regular, unprotected intercourse, increases significantly. This underscores the need for a holistic approach to addressing obesity and its implications for fertility, encompassing lifestyle modifications and specialized medical care [11]. The rising prevalence of obesity has contributed to a global health crisis and presented a formidable challenge to those aspiring to start families. The intricate connection between obesity and infertility underscores the importance of addressing weight management as a fundamental aspect of reproductive health, highlighting the need for prevention and intervention strategies to mitigate these adverse effects on fertility and promote healthier outcomes for individuals and couples alike [5].

Importance of IVF as a Fertility Treatment Option

Overcoming ovulatory disorders: Obesity is often intricately linked with ovulatory disorders, such as PCOS, which can hinder natural conception. IVF is particularly advantageous as it effectively bypasses these ovulatory challenges. Through IVF, eggs are directly harvested from the ovaries, ensuring a steady supply of eggs for fertilization, thereby circumventing the irregular ovulation associated with PCOS [12]. Controlled environment: Obesity can lead to hormonal imbalances that disrupt the reproductive process. IVF provides a controlled laboratory environment where factors influencing fertilization can be meticulously managed. This precise control helps mitigate some of the challenges posed by obesity-related hormonal fluctuations, enhancing the chances of successful fertilization and embryo development [13-14]. Preimplantation genetic testing: Obesity can introduce additional health concerns that may be inherited by offspring. IVF uniquely offers the opportunity for preimplantation genetic testing (PGT), a procedure that allows for the screening of embryos before their transfer. This valuable tool enables the selection of embryos with the highest likelihood of success and can help identify and exclude embryos carrying specific genetic conditions. For obese patients, this aspect of IVF can be particularly relevant, as it offers a means to reduce the likelihood of passing on inherited health risks to their children [15].

Anesthetic evaluation of obese women patients

Preoperative Assessment and Patient History

Evaluation of comorbidities: The preoperative assessment of comorbid conditions is paramount, particularly in obese patients undergoing surgical procedures. Obesity is often accompanied by a higher prevalence of medical comorbidities, necessitating a thorough evaluation to manage patient risks comprehensively. Conditions such as diabetes, hypertension, cardiovascular disease, and obstructive sleep apnea (OSA) are frequently encountered in the obese population and can significantly influence anesthesia management. Assessing these comorbidities' nature, severity, and control is critical in determining the patient's perioperative risk profile. This assessment is a foundation for tailoring anesthetic strategies and optimizing patient safety [16]. Medication history: A meticulous review of the patient's medication history is indispensable to the anesthetic evaluation process. This assessment goes beyond merely identifying the medications a patient is currently taking; it encompasses a nuanced understanding of how these medications might interact with anesthesia and surgical agents. Particular attention should be directed toward medications impacting anesthetic management, such as anticoagulants, antihypertensive drugs, and substances that interact with anesthesia agents. The decision-making process regarding continuing, modifying, or temporarily discontinuing specific medications should be conducted in close collaboration with the patient's primary care physician or relevant specialists. This collaborative approach ensures that the patient's medical needs are met while minimizing potential perioperative risks [17]. Allergies and sensitivities: Identifying allergies and sensitivities is pivotal in preoperative assessment, primarily safeguarding patient well-being during surgery. It is imperative to uncover any allergies or sensitivities the patient may have to medications, latex, or other materials commonly employed in the operating room environment. This information is critical for tailoring the anesthetic regimen, selecting appropriate drugs and equipment, and preventing allergic reactions or adverse events during surgery. Rigorous documentation of these allergies and sensitivities is indispensable, ensuring that the entire perioperative team is well-informed and can collaborate effectively to provide a safe and uneventful surgical experience for the patient [18].

Physical Examination in Obese Patients

Assessment of airway anatomy: Obese patients often present unique challenges related to their airway anatomy. Limited neck mobility, short and thick necks, and excess tissue in the oropharyngeal and supraglottic areas may characterize their airways. These anatomical variations can complicate airway management significantly during anesthesia induction and intubation. Anesthesiologists must carefully consider these factors and employ suitable airway management strategies, such as specialized equipment or alternative airway approaches, to mitigate potential difficulties and ensure safe ventilation [19]. Evaluation of cardiovascular status: Obesity is closely associated with an increased risk of cardiovascular diseases, including hypertension, coronary artery disease, and congestive heart failure. As such, a meticulous assessment of the patient's cardiovascular status is imperative. This evaluation may include ECG to identify any arrhythmias or baseline cardiac abnormalities and echocardiography in cases where a more comprehensive assessment of cardiac function is warranted. Understanding the patient's cardiovascular health is crucial for tailoring anesthesia plans and selecting appropriate medications to optimize hemodynamic stability during surgery [20]. Assessment of respiratory function: Obesity is a significant risk factor for OSA, characterized by repetitive interruptions in breathing during sleep. Consequently, obese individuals are at a heightened risk of experiencing perioperative respiratory complications. To address this risk, anesthesiologists should consider assessing respiratory function by conducting pulmonary function tests and evaluating oxygenation status. These assessments provide valuable insights into lung compliance, oxygen exchange capacity, and underlying respiratory conditions, guiding the choice of ventilation strategies and monitoring techniques to safeguard respiratory well-being during surgery [21].

Laboratory Tests and Investigations

Complete blood count (CBC): Rather than delving into the definition of a CBC, it is pertinent to elucidate the changes observed in CBC counts within the obese population. Such insights can prove invaluable for healthcare professionals, especially physicians, as they navigate the challenges presented by obesity. In this context, the CBC becomes an essential tool that scrutinizes various blood components, including RBCs, WBCs, and platelets. Each component within the CBC bears crucial information for anesthesia providers during the preoperative evaluation process. For instance, the RBC count and hemoglobin levels are pivotal in assessing the patient's oxygen-carrying capacity. Low values may indicate anemia, a condition that, if left unaddressed, can adversely impact tissue oxygenation during surgery. WBC counts offer insights into the patient's immune status. Deviations from the normal range may indicate underlying infections or inflammatory conditions, necessitating preemptive action before surgery. Platelet counts, vital for normal blood clotting, help in assessing bleeding risks and guiding decisions regarding blood transfusions or medications such as anticoagulants. By examining these hematological parameters and understanding their variations within the obese population, anesthesia providers can make informed decisions to mitigate bleeding risks, optimize oxygen delivery, and ensure a safer perioperative experience [22]. Coagulation profile (PT, aPTT, INR): Rather than delving into an explanation of these specific pathological tests, it is imperative to underscore the alterations observed in the coagulation profiles of obese individuals. These tests, including prothrombin time (PT), activated partial thromboplastin time (aPTT), and international normalized ratio (INR), are vital tools for assessing bleeding risks and ensuring effective coagulation during surgery. In obese patients, these assessments take on heightened significance due to the potential changes in their coagulation profiles, attributable to factors such as increased adipose tissue and alterations in clotting factor metabolism. Obese patients often exhibit variations in their clotting ability, necessitating scrutiny of their coagulation parameters. Anesthesia providers rely on these parameters to make crucial decisions regarding hemostasis and anticoagulation management during surgery. Precise monitoring and adjustments of anticoagulant medications and strategies are guided by the unique coagulation information obtained from obese patients, ultimately minimizing the risks associated with excessive bleeding and thrombotic events [23]. Electrolyte panel: An electrolyte panel offers insights into the patient's electrolyte balance, which is essential for maintaining normal physiological functions, including nerve conduction, muscle contraction, and cardiac rhythm. This assessment measures levels of critical ions such as sodium, potassium, and calcium. Disturbances in electrolyte balance can lead to severe complications, including cardiac arrhythmias and neuromuscular dysfunction, which can have dire consequences during surgery. The data obtained from this panel informs anesthesia providers of any imbalances, enabling timely interventions to correct electrolyte abnormalities, optimize patient stability, and minimize perioperative risks [24]. Renal and hepatic function tests: Rather than dwelling on the definitions of these tests, it is essential to focus on the specific changes observed in the pathological database of obese populations. This perspective provides valuable insights for healthcare providers, as it impacts the safe administration of anesthesia. Renal function tests, such as serum creatinine and glomerular filtration rate (GFR), are critical in assessing the kidneys' ability to filter and eliminate waste products and anesthetic agents. Notably, abnormalities in renal function can significantly affect medication dosing and the selection of appropriate anesthetic agents for obese individuals. Similarly, hepatic function tests, encompassing liver enzymes and bilirubin levels, offer crucial information about the liver's capability to metabolize medications and synthesize essential clotting factors. In the context of obesity, impaired hepatic function can have a profound impact on anesthesia management, potentially necessitating dose adjustments and medication selection modifications. By examining the specific variations in renal and hepatic function within the obese population, anesthesia providers can tailor their approach to ensure the safe and effective administration of anesthetics, thus minimizing the risks associated with drug accumulation and potential toxicity [25]. Glycemic control assessments (especially in diabetic patients): For obese patients with diabetes or impaired glucose control, glycemic assessments are paramount. These assessments encompass fasting blood glucose and HbA1c levels, offering valuable insights into the patient's diabetic status and glycemic control. Maintaining tight glycemic control during the perioperative period is essential to prevent complications such as hyperglycemia, which can lead to impaired wound healing and increased infection risks, or hypoglycemia, which can result in adverse events such as altered mental status and cardiac arrhythmias. Anesthesia providers use these assessments to guide glucose management strategies, including insulin administration, to ensure stable and well-controlled blood glucose levels throughout surgery [26]. Arterial blood gas (ABG) analysis: ABG analysis comprehensively assesses a patient's oxygenation, acid-base balance, and respiratory function. This analysis provides essential data for anesthesia providers, especially in obese individuals who are at an increased risk of respiratory complications. ABG analysis informs decisions regarding ventilation strategies and settings, ensuring oxygenation and carbon dioxide elimination are effectively maintained during surgery. It also aids in identifying potential respiratory acidosis or alkalosis, which can guide interventions to restore normal acid-base equilibrium. By closely monitoring these parameters, anesthesia providers can proactively address respiratory challenges, prevent perioperative respiratory distress, and optimize patient oxygenation and ventilation [27].

Anesthetic Risk Stratification

Following the comprehensive preoperative assessment, anesthetic risk stratification should be performed. This involves categorizing patients into risk groups based on their comorbidities, physical status, and the nature of the surgical procedure. Standard classification systems include the American Society of Anesthesiologists (ASA) Physical Status Classification and the Charlson Comorbidity Index [28]. Anesthetic risk stratification guides the selection of appropriate anesthetic techniques and perioperative management strategies for obese patients. High-risk individuals may require more intensive monitoring, specialized equipment, and close collaboration with other healthcare providers to optimize their perioperative care [29].

Anesthetic management strategies

Preoperative Optimization

Weight loss and lifestyle modification: Preoperative weight loss and lifestyle modification are proactive and potentially transformative strategies for obese patients embarking on elective surgery, including IVF procedures. These initiatives are driven by the recognition that obesity can engender complex health concerns and perioperative challenges. Here, we delve deeper into the multifaceted aspects of preoperative weight loss and lifestyle modification, elucidating their manifold benefits [30]. Improvements in comorbidities: Obesity often accompanies a constellation of comorbid conditions, from diabetes to hypertension and OSA. Weight loss efforts, even modest ones, can yield substantial improvements in these conditions. As excess weight diminishes, blood pressure tends to normalize, blood glucose control improves, and the severity of sleep apnea can attenuate. These changes are pivotal as they help reduce the perioperative risks associated with these comorbidities [31]. Mitigation of surgical risks: Obesity is a known risk factor for surgical complications, encompassing concerns like wound infections, deep vein thrombosis (DVT), and cardiovascular events. Preoperative weight loss can mitigate some surgical risks by reducing adipose tissue associated with chronic inflammation and metabolic dysregulation. Additionally, as weight loss often leads to improved fitness levels, patients may experience enhanced cardiovascular function, better oxygen delivery, and an overall reduction in the strain placed on their bodies during surgery [32]. Enhancement of IVF success: Beyond surgery, weight loss can have profound implications for couples seeking fertility assistance through IVF. Obesity is intrinsically linked to infertility, with irregular menstrual cycles and hormonal imbalances commonly affecting obese women. By promoting more regular ovulatory cycles and hormonal balance, weight loss can increase the chances of successful IVF outcomes. Therefore, for those navigating both infertility and obesity, preoperative weight loss can offer the dual benefits of improving surgical outcomes and bolstering fertility prospects [33]. Collaboration with healthcare experts: Achieving sustainable weight loss necessitates a collaborative approach involving healthcare professionals with nutrition, dietetics, and exercise physiology expertise. Dietitians and nutritionists can help patients develop customized dietary plans that promote healthy weight reduction while ensuring essential nutrient intake. Exercise specialists can design safe and effective physical activity regimens tailored to individual fitness levels and capabilities. This multidisciplinary collaboration empowers patients to embark on a weight loss journey that is both realistic and sustainable, extending well beyond the immediate preoperative period [34].

Glycemic Control

Crucial for surgical success: Elevated blood glucose levels in patients with diabetes can heighten the risk of surgical complications, including impaired wound healing, increased infection susceptibility, and cardiovascular events. Achieving and maintaining optimal glycemic control before surgery can mitigate these risks, promoting a smoother and safer perioperative experience [35]. Implications for IVF: For obese individuals undergoing IVF, glycemic control assumes even greater importance. Diabetes, if uncontrolled, can adversely affect fertility and pregnancy outcomes. Elevated blood glucose levels can disrupt hormonal balance, impair ovarian function, and increase the risk of complications during pregnancy. Therefore, meticulous glycemic control before embarking on an IVF journey is pivotal for optimizing the chances of a successful outcome [5]. Close coordination with specialists: Achieving optimal glycemic control often necessitates close collaboration with healthcare specialists, such as endocrinologists or diabetologists. These experts can assess the patient's diabetic status, review current medication regimens, and make necessary adjustments to ensure stable blood glucose levels during the perioperative period. Medication dosages may need to be modified, and specific protocols for glycemic management before, during, and after surgery may be established to maintain glucose homeostasis [36].

Anesthetic choices

General Anesthesia vs. Regional Anesthesia

Table 2 summarizes the key considerations in selecting the most appropriate anesthesia approach for IVF procedures. It contrasts the advantages and preferences associated with general and regional anesthesia and highlights the relevance of anesthetic risks, particularly in the context of obese patients. This table is a valuable reference for healthcare professionals when making informed decisions about anesthesia methods for IVF procedures.

**Table 2:** Considerations for selecting an anesthetic approach in IVF procedures. IVF: In vitro fertilisation.

Consideration	General Anesthesia	Regional Anesthesia
Procedure Specificity [37]	Appropriate for IVF procedures that require complete unconsciousness or involve a relatively lower abdominal surgical approach.	Favored in select IVF cases, reducing airway-related complications and improving postoperative pain control.
Patient Preferences [38]	Some patients may prefer complete sedation or unconsciousness.	Some patients may prefer to remain conscious or semi-conscious during the procedure.
Anesthetic Risks [39]	May be preferred for obese patients with severe respiratory compromise or heightened risk of aspiration.	May be preferred for obese patients with factors like increased neck circumference, reduced lung compliance, and potential airway difficulties.

Sedation Options

Patient comorbidities: Comorbid conditions like cardiovascular disease or OSA should be carefully considered when choosing sedation options. Some medications used for sedation may have hemodynamic effects or exacerbate respiratory compromise, necessitating a tailored approach that minimizes these risks. Additionally, anesthetic providers must monitor vital signs and promptly address adverse reactions [40]. Airway management: The choice of sedation should be compatible with the patient's airway management needs. In obese patients with potentially challenging airways, anesthetic providers should be prepared to swiftly transition to more profound levels of sedation or general anesthesia if airway maintenance becomes problematic [41]. Recovery considerations: The duration and nature of IVF procedures may influence the choice of sedation. Shorter procedures may benefit from lighter sedation techniques, allowing quicker recovery and reduced postoperative drowsiness. Conversely, more prolonged procedures may necessitate more profound sedation to ensure patient comfort and cooperation throughout the process [42].

Equipment considerations

Specially Designed Operating Room Tables and Equipment

Bariatric operating tables: Bariatric operating tables are designed to support the weight and dimensions of obese patients effectively. They offer enhanced stability and durability, which is essential during surgical procedures. These tables are more comprehensive and feature robust mechanisms for adjusting patient positioning. This ensures that patients are adequately supported, minimizing the risk of complications related to positioning, such as pressure ulcers or nerve injuries [43]. More comprehensive surgical instruments: Surgeons require a range of instruments suitable for the unique anatomical characteristics of obese patients. This includes longer and wider surgical instruments, specialized retractors, and trocars designed to navigate the increased tissue depth associated with obesity. These instruments enable surgeons to perform procedures safely and efficiently, reducing the risk of tissue damage or complications [44]. Appropriately sized anesthesia equipment: Anesthesia providers must have access to appropriately sized equipment to ensure patient stability and accessibility. This encompasses more oversized blood pressure cuffs, appropriately sized tourniquets, and adequately sized intravenous catheters. Ensuring these items are readily available in the operating room helps streamline the anesthesia process and prevents the need for last-minute adjustments or replacements [45].

Airway management challenges

Advanced Airway Management Skills

Anesthesia providers should possess advanced airway management skills, allowing them to adapt to the anatomical nuances of obese patients. This includes expertise in various intubation techniques and strategies for optimizing ventilation. Providers should be prepared to handle scenarios where conventional intubation methods may prove challenging [41].

Video Laryngoscopes

Video laryngoscopes are invaluable tools in managing obese patients with difficult airways. These devices incorporate a camera at the tip of the laryngoscope blade, offering a clear view of the airway and vocal cords. This visual aid enhances the accuracy of intubation attempts, even in cases where direct laryngoscopy may be challenging due to anatomical factors [46].

Supraglottic Airway Devices

Supraglottic airway devices, such as the laryngeal mask airway (LMA), are crucial in securing the airway in obese patients. These devices are inserted above the vocal cords and are particularly useful in scenarios where endotracheal intubation is challenging. LMAs provide a secure airway for positive pressure ventilation and can be a bridge to definitive airway management if needed [47].

Comprehensive Training

Anesthesia providers should undergo comprehensive training in advanced airway management tools and techniques, especially when caring for obese patients. Regular simulation exercises and ongoing education help maintain proficiency and ensure readiness to address airway challenges effectively [48].

Medication dosing in obese patients

Induction Agents

Adjusting for altered pharmacokinetics: Obesity is associated with significant drug distribution and metabolism changes. In particular, the increased adipose tissue content and expanded volume of distribution can lead to prolonged drug effects. As a result, dosing of induction agents like propofol and etomidate should be cautiously approached in obese patients [49].

Consideration of ideal body weight or lean body mass: To prevent the risk of overdosing and subsequent prolonged sedation or respiratory depression, anesthesia providers often base dosing on ideal body weight or lean body mass rather than total body weight in obese patients. Ideal body weight is calculated based on a healthy BMI, which allows for a more accurate alignment of drug dosing with the patient's physiological characteristics [50].

Maintenance Agents

Careful titration based on patient response: Maintenance agents, including inhalational anesthetics and intravenous infusions, require precise titration based on the patient's response. Continuous monitoring of the depth of anesthesia is essential throughout the procedure. Technologies like bispectral index (BIS) monitoring provide real-time feedback on the patient's level of consciousness, enabling anesthesia providers to adjust maintenance agent dosages as needed [51].

Balancing anesthesia depth: The primary goal is to achieve a balanced anesthetic state that ensures patient comfort while minimizing the risks associated with excessive or inadequate anesthesia depth. Overly deep anesthesia can result in delayed emergence, whereas insufficient depth may lead to intraoperative awareness [51].

Neuromuscular Blocking Agents

Higher dosing requirements: Due to the increased volume of distribution in obese patients, higher initial doses of neuromuscular blocking agents (NMBAs) like rocuronium or vecuronium may be necessary to achieve the desired level of muscle relaxation for surgical procedures. This adjustment is crucial to ensure the effectiveness of NMBA-induced paralysis [52].

Monitoring neuromuscular blockade: Train-of-four (TOF) monitoring is paramount when administering NMBAs to obese patients. TOF monitoring assesses the depth of neuromuscular blockade, aiding anesthesia providers in precise dose titration. This approach ensures that the degree of paralysis aligns with the requirements of the surgical procedure, preventing both inadequate blockade and excessive paralysis. Continuous monitoring continues throughout the surgery to guide the administration of reversal agents when necessary, facilitating a smooth recovery [53].

Analgesics

Weight-adjusted dosing: Pain management is crucial for obese patients during and after surgery. The dosing of analgesics, whether opioids or non-opioid alternatives, should be adjusted to account for weight-related factors. This adjustment ensures that analgesic effects are adequate to control pain effectively while minimizing the risk of overdose [54].

Multimodal analgesia: In obese patients, a multimodal analgesic approach is often favored to reduce reliance on opioids and mitigate their associated side effects, including respiratory depression and postoperative nausea and vomiting. This approach may encompass non-opioid analgesics, regional anesthesia techniques, and adjunct medications like non-steroidal anti-inflammatory drugs (NSAIDs). By combining multiple analgesic modalities, anesthesia providers can enhance pain control and improve the overall safety profile of pain management in obese patients [55].

Ventilation strategies

Risk of Hypoventilation and Respiratory Complications

Increased vulnerability: Obese patients have unique anatomical and physiological characteristics that make them more susceptible to hypoventilation, oxygen desaturation, and postoperative respiratory complications. These challenges stem from decreased lung compliance, reduced functional residual capacity, and a propensity for upper airway obstruction [56].

Positive end-expiratory pressure (PEEP): PEEP is often employed to address these concerns. PEEP helps maintain lung volume by preventing alveolar collapse during expiration. This technique aids in improving oxygenation and mitigating atelectasis, which is a common issue in obese individuals during anesthesia and surgery [57].

Monitoring oxygen saturation: Continuous monitoring of oxygen saturation (SpO2) is essential throughout the surgical procedure to promptly detect any declines in oxygen levels. Obese patients may experience rapid desaturation during periods of hypoventilation, making vigilant monitoring crucial for patient safety [58].

Monitoring end-tidal CO2 (ETCO2): Continuous monitoring of end-tidal carbon dioxide (ETCO2) levels, known as capnography, is a fundamental component of anesthesia for obese patients. ETCO2 monitoring provides real-time feedback on ventilation and allows anesthesia providers to detect hypoventilation early. Rising ETCO2 levels can be an early sign of inadequate ventilation, enabling timely intervention to prevent hypercapnia and associated complications [59].

Elevation of the Head of the Bed

Improved ventilation: During surgery, elevating the head of the bed in obese patients can help improve ventilation. This adjustment assists in reducing the risk of upper airway obstruction, which is more prevalent when patients are supine. Proper positioning is critical to optimize lung mechanics and maintain effective ventilation [60].

Positioning and Pressure Sore Prevention

Proper patient positioning is crucial to ensure access to the surgical site and prevent pressure sores, especially in obese patients with limited mobility. Pressure-relieving pads and regular position changes should be employed to minimize the risk of skin complications during prolonged procedures. A thorough understanding of these anesthetic management strategies tailored to obese patients is essential for anesthesia providers and healthcare teams to ensure safe and effective care during IVF procedures [61].

Intraoperative and postoperative considerations

Hemodynamic Monitoring

Continuous surveillance of hemodynamics is paramount in the care of obese patients undergoing IVF procedures. A higher prevalence of cardiovascular comorbidities, such as hypertension, coronary artery disease, and heart failure, often accompanies obesity. As a result, meticulous attention to hemodynamic parameters is essential to detect and manage potential perioperative cardiovascular issues [62].

Blood Pressure Monitoring

Close and continuous blood pressure monitoring, including systolic and diastolic measurements, is crucial. Automated non-invasive blood pressure cuffs should be appropriately sized to ensure accurate readings. This ongoing assessment enables anesthesia providers to detect and respond promptly to changes in blood pressure, whether due to surgical manipulation, anesthesia effects, or underlying cardiovascular conditions [63].

Heart Rate Monitoring

Continuous heart rate monitoring is vital to detect any arrhythmias or hemodynamic fluctuations promptly. Anesthesia providers must be prepared to address bradycardia or tachycardia as needed during the surgery, ensuring that the patient remains stable throughout the procedure [64].

Cardiac Output Assessment

In certain high-risk or complex cases, assessing cardiac output may be necessary. This can be achieved through various techniques, including thermodilution or less invasive methods like pulse contour analysis. Monitoring cardiac output provides valuable insights into the patient's circulatory status and helps guide fluid management and vasopressor/inotropic support when indicated [65].

Temperature management

Maintaining normothermia is of paramount importance in the intraoperative care of obese female patients undergoing IVF. These patients present unique considerations due to their larger body surface area and altered thermoregulation. Such factors elevate their susceptibility to perioperative hypothermia, a condition that can have detrimental consequences, including coagulopathy, heightened vulnerability to surgical site infections, and increased cardiovascular stress [66].

To safeguard the well-being of these patients, it is imperative to employ active warming measures specifically tailored to their needs. One widely utilized method involves the use of forced air warming blankets, which deliver warm air directly to the patient's body, helping to counteract the challenges posed by their altered thermoregulation. Furthermore, warmed intravenous fluids can be employed as an effective means to maintain the core body temperature within the desired range for this patient population. Continuous temperature monitoring throughout the IVF procedure is essential, enabling immediate intervention in case of any deviations from the optimal normothermic range [67].

Fluid management

In fluid management during surgical procedures, particularly for obese patients, two crucial considerations demand attention. Firstly, obese patients are at a heightened risk of overhydration, which can result in severe complications, including pulmonary edema and cardiovascular strain. Therefore, healthcare providers must exercise caution when administering fluids to this patient population. Implementing individualized fluid management strategies tailored to each patient's unique needs is essential. These strategies should be guided by hemodynamic monitoring, and in some cases, invasive measures might be required to ensure precise fluid balance and prevent overhydration [29].

Secondly, preventing complications like pulmonary edema is paramount when caring for obese surgical patients. To achieve this, it is imperative to avoid excessive fluid administration. Instead, the focus should be on optimizing oxygenation levels. Continuous monitoring of oxygen saturation and end-tidal CO2 and a vigilant assessment for early signs of fluid overload, such as crackles during auscultation, must be integral to the surgical team's protocol. By adhering to these meticulous fluid management and monitoring practices, healthcare providers can minimize the risks associated with overhydration and pulmonary edema, ultimately ensuring safer surgical outcomes for obese patients [68].

Postoperative considerations

Postoperative care for obese patients demands a comprehensive and tailored approach to ensure their safety and well-being during recovery. Firstly, transitioning from the operating room to the recovery room necessitates vigilant monitoring. Healthcare providers must closely observe vital signs, oxygen saturation levels, and consciousness levels and remain alert to any signs of postoperative complications, such as respiratory distress or cardiovascular instability. This heightened level of surveillance is crucial in the immediate postoperative phase to promptly address any issues that may arise [16].

Effective pain management is another critical facet of postoperative care for obese patients. Recognizing that they may require higher doses of analgesics due to altered pharmacokinetics, healthcare teams should adopt multimodal analgesic approaches. These approaches should incorporate non-opioid options to minimize the potential for opioid-related side effects while optimizing pain control and ensuring patient comfort [69].

Early mobilization is a cornerstone of postoperative care for obese individuals. Promptly getting patients up and moving helps mitigate the risk of complications associated with immobility, such as atelectasis (lung collapse) and DVT. Collaboration between physical therapists and nursing staff is vital in facilitating early ambulation and encouraging deep breathing exercises, which can significantly contribute to a smoother recovery [70].

Obese patients are particularly susceptible to postoperative complications, notably atelectasis and DVT. Reduced lung compliance and hypercoagulability make them prone to these issues. Therefore, preventive measures should be integrated into the postoperative care plan. This may include using incentive spirometry to promote lung expansion, early ambulation to improve circulation and lung function, and pharmacological thromboprophylaxis to minimize the risk of DVT formation. By implementing these strategies and maintaining a vigilant postoperative care regimen, healthcare providers can help obese patients recover safely and minimize the potential for complications [71].

Special considerations for IVF

Timing of Anesthesia and IVF Procedures

The timing of anesthesia in conjunction with IVF procedures is a critical and intricately coordinated aspect of patient care. Firstly, anesthesia providers must establish a close and seamless collaboration with the IVF team to ensure that anesthesia induction and surgery are perfectly synchronized with the patient's specific IVF protocol. This synchronization is vital to optimize the timing of crucial IVF procedures, including oocyte retrieval, insemination, and embryo transfer, thereby maximizing the chances of a successful outcome [72].

Oocyte retrieval stands out as a pivotal moment within the IVF process. To ensure patient comfort and minimize discomfort during the transvaginal follicular aspiration, anesthesia should be administered before this procedure. The timing and administration of anesthesia at this stage are critical to ease the patient's experience and allow the IVF team to perform the retrieval smoothly and efficiently [73].

Regarding embryo transfer, the anesthesia approach may differ from oocyte retrieval. In many cases, patients may not require general anesthesia for this procedure. Instead, conscious sedation or regional anesthesia techniques may be more appropriate and well-tolerated by patients. The choice of anesthesia should consider the patient's preferences, comfort, and any specific medical considerations. This personalized approach ensures that the patient remains as relaxed as possible during this crucial step of the IVF process, contributing to a positive overall experience [74].

Implications of Obesity on IVF Success Rates

Reduced oocyte quality: One of the primary consequences of obesity in the context of IVF is the potential for reduced oocyte (egg) quality in obese women. This decline in oocyte quality can manifest as abnormalities, such as irregular chromosome structure and spindle formation. As a result, obese individuals may experience lower fertilization rates and reduced embryo implantation rates during IVF. These challenges can pose significant obstacles to achieving a successful pregnancy [75].

Altered hormonal profiles: Obesity disrupts hormonal profiles in several ways. Notably, it often leads to increased insulin resistance, a condition in which cells become less responsive to the effects of insulin. Additionally, obesity can alter sex hormone levels, causing elevated estrogen and androgen levels while reducing levels of sex hormone-binding globulin (SHBG). These hormonal imbalances can negatively impact ovarian function, potentially leading to irregular menstrual cycles and suboptimal reproductive outcomes [76].

Increased risk of miscarriage: Obese patients undergoing IVF are at an elevated risk of experiencing miscarriages. This heightened risk emphasizes the importance of preoperative health and weight management optimization. Furthermore, during pregnancy, obese individuals require close monitoring to promptly identify and manage any complications that may arise, given the increased risk associated with their condition [77].

Diminished response to medications: Obesity can result in a diminished response to fertility medications used in IVF, particularly gonadotropins. Due to altered pharmacokinetics, obese patients may require higher doses of these medications to stimulate ovarian follicular development effectively. Tailoring medication dosages to individual patient needs is crucial in optimizing IVF outcomes [78].

Increased risk of complications: Obesity is associated with a heightened risk of complications during IVF procedures. Notably, obese individuals may be at an increased risk of developing ovarian hyperstimulation syndrome (OHSS), characterized by enlarged ovaries and fluid accumulation in the abdomen and chest. Additionally, they may face an elevated risk of thromboembolic events, such as blood clots, during the IVF process. Careful management and monitoring are essential to mitigate these risks and ensure the safety and success of IVF procedures in obese patients [79].

Emotional and Psychological Support for Obese Patients Undergoing IVF

Patient counseling: Preoperative counseling tailored to IVF's emotional and psychological aspects is essential for obese patients. It's important to create a safe and non-judgmental space where patients can openly discuss their anxieties, body image concerns, and expectations related to fertility treatment. Addressing these issues early can help patients better cope with the emotional rollercoaster often accompanying IVF [80].

Support groups: Obese patients undergoing IVF can benefit immensely from participating in support groups or counseling sessions for individuals facing similar challenges. These groups provide a supportive environment where patients can share their experiences, express their emotions, and receive advice and encouragement from peers who have undergone similar journeys. Connecting with others who understand their unique struggles can be comforting and empowering [81].

Multidisciplinary approach: A holistic approach involving a team of healthcare professionals is crucial. This multidisciplinary team may include psychologists, social workers, dietitians, and reproductive specialists collaborating to provide comprehensive support. Psychologists and social workers can help patients address the emotional impact of infertility and obesity on their mental health, offering coping strategies and emotional resilience tools [82].

Education: Providing patients with accurate and detailed information about the relationship between obesity, IVF, and fertility outcomes is key. Clear and open communication helps patients make informed decisions and manage their expectations realistically. Educating patients about the specific challenges they may face due to obesity and the potential impact on IVF success can help alleviate anxiety and uncertainty [83].

Challenges and future directions

Ongoing Research in the Field

Obesity-related factors: Researchers are delving into specific obesity-related factors that can influence fertility and IVF outcomes. This includes studying adipokines (hormones produced by fat tissue), inflammatory markers, and genetic predispositions that may impact reproductive health. Understanding the molecular mechanisms underlying these factors can provide insights into potential interventions to improve fertility and IVF success rates in obese patients [84].

Anesthetic techniques: Research in this area evaluates the safety and efficacy of various anesthetic techniques tailored to obese patients undergoing IVF. This may include investigating the benefits and risks of regional anesthesia options, optimizing sedation strategies, and developing drug dosing protocols that account for the altered pharmacokinetics and pharmacodynamics often seen in obese individuals. Finding the safest anesthesia approach can improve patient comfort and procedural outcomes [29].

IVF protocols: Modified IVF protocols designed to address the unique challenges presented by obesity are a critical area of research. Scientists and clinicians are working on optimizing ovarian stimulation regimens to improve oocyte quality and quantity in obese women. Additionally, researchers aim to develop protocols that reduce the risk of complications like ovarian hyperstimulation syndrome (OHSS) in this patient population, ensuring safer and more successful IVF cycles [85].

Long-term outcomes: Research efforts also extend to studying the long-term outcomes of obese patients and their offspring following IVF. This includes assessing the impact of maternal obesity on maternal and neonatal health and investigating potential epigenetic changes that may occur during IVF procedures. Understanding the lasting effects of IVF in obese patients is crucial for providing comprehensive healthcare and counseling to these individuals and their families [86].

Emerging Technologies and Techniques

Precision medicine: The application of precision medicine in IVF involves tailoring treatment protocols and anesthesia management to each patient's specific genetic and metabolic characteristics. By analyzing an individual's genetic and metabolic profile, healthcare providers can make more informed decisions regarding fertility medications, dosages, and anesthesia techniques. This personalized approach can optimize treatment outcomes and minimize risks for obese patients, who often exhibit unique physiological variations [87]. Robotic-assisted surgery: Robotic-assisted surgery is being explored as a valuable tool in IVF procedures, particularly for cases requiring laparoscopic interventions. The precision and dexterity offered by robotic systems can enhance the surgeon's ability to perform delicate procedures, such as oocyte retrieval or tubal surgeries, with greater accuracy. This can reduce surgical complications and improve outcomes for obese IVF patients [88]. Pharmacogenomics: Pharmacogenomics is a burgeoning field investigating the genetic factors influencing individual medication responses. By analyzing a patient's genetic makeup, healthcare providers can predict how they will respond to anesthesia drugs and fertility medications. This information can be used to tailor treatment plans, ensuring that obese patients receive the most effective and safest medications at optimal dosages, thereby enhancing their IVF experience [89]. Artificial intelligence (AI): AI technologies are increasingly employed in IVF care. AI algorithms can analyze extensive patient data, including obesity-related factors, to predict IVF success rates. By considering a patient's unique characteristics, AI can provide more accurate prognoses and guide treatment decisions. Additionally, AI can optimize anesthesia dosing and management in real-time, adjusting drug administration based on continuous monitoring of patient responses and enhancing safety and comfort during IVF procedures for obese individuals [90].

The Role of Multidisciplinary Teams in Managing Obese Patients Undergoing IVF

Collaboration: Effective care for obese patients undergoing IVF requires close collaboration among healthcare specialists. This includes anesthesiologists, reproductive endocrinologists, bariatric specialists, nutritionists, psychologists, and other relevant professionals. These experts work together to develop integrated care plans that address both the medical and psychosocial aspects of obesity and fertility treatment. This collaborative effort ensures that all aspects of a patient's health and well-being are considered [91]. Tailored care: Recognizing that obese patients have unique needs, multidisciplinary teams design individualized care pathways for these specific requirements. This includes preoperative optimization, which may involve weight management strategies, addressing comorbidities, and optimizing reproductive health. During the IVF procedure, tailored anesthesia and medication protocols are implemented. Postoperative care and ongoing fertility treatment plans are customized to address the patient's evolving needs [92]. Patient education: Multidisciplinary teams play a crucial role in educating obese patients about the relationship between obesity and fertility and the potential impact of obesity on IVF success rates. Patients are provided with comprehensive information about lifestyle modifications, weight management strategies, and the importance of adherence to treatment plans. Education empowers patients to make informed decisions about their care and plays a significant role in achieving better outcomes [93]. Shared decision-making: In a multidisciplinary approach, shared decision-making is emphasized. Healthcare providers collaborate with patients to ensure treatment plans align with their values, preferences, and goals. This patient-centered approach fosters trust and open communication, allowing patients to actively participate in their care and make choices that best suit their circumstances [94].

## Conclusions

In conclusion, the anesthetic evaluation and management of obese patients undergoing IVF procedures is multifaceted and demands precision, empathy, and a multidisciplinary approach. This comprehensive review has underscored the significance of addressing obesity's impact on IVF success rates, the importance of tailored anesthesia and perioperative care, and the need for emotional support throughout the IVF journey. Obesity poses unique medical and psychological challenges, necessitating a holistic approach that bridges the realms of anesthesia, reproductive medicine, and patient well-being. As we move forward, ongoing research, emerging technologies, and collaborative efforts among healthcare providers promise to enhance further the care of obese individuals seeking fertility treatments. By refining our practices and understanding, we can empower these patients to achieve the dream of parenthood while ensuring their safety, comfort, and overall success.
